# Better and fulfilling healthcare at lower costs: The need to manage health systems as complex adaptive systems

**DOI:** 10.12688/f1000research.19414.1

**Published:** 2019-06-05

**Authors:** Joachim P. Sturmberg, Johannes Bircher

**Affiliations:** 1 School of Medicine and Public Health, Faculty of Health and Medicine, University of Newcastle, Holgate, NSW, 2250, Australia; 2 International Society for Systems and Complexity Sciences for Health, Waitsfield, VT, USA; 3 Hepatology Department of Biomedical Research, University of Bern, Bern, Switzerland

**Keywords:** Healthcare costs, Healthcare financing, Healthcare as complex adaptive system, Sense or purpose of healthcare, Definition of health, Healthcare organization, Norms in healthcare, Complex adaptive systems, System dynamics, Philosophy of medicine

## Abstract

Rising healthcare costs are major concerns in most high-income countries. Yet, political measures to reduce costs have so far remained futile and have damaged the best interests of patients and citizen. We therefore explored the possibilities to analyze healthcare systems as a socially constructed complex adaptive system (CAS) and found that by their very nature such CAS tend not to respond as expected to top-down interventions. As CAS have emergent behaviors, the focus on their drivers – purpose, economy and behavioral norms – requires particular attention. First, the importance of understanding the purpose of health care as improvement of health and its experience has been emphasized by two recent complementary re-definitions of health and disease. The economic models underpinning today’s healthcare – profit maximization – have shifted the focus away from its main purpose. Second, although economic considerations are important, they must serve and not dominate the provision of healthcare delivery. Third, expected health professionals’ behavioral norms – to first consider the health and wellbeing of patients – have been codified in the universally accepted Declaration of Geneva 2017. Considering these three aspects it becomes clear that complex adaptive healthcare systems need mindful top-down/bottom-up leadership that supports the nature of innovation for health care driven by local needs. The systemic focus on improving people’s health will then result in significant cost reductions.

## Introduction

In high income countries healthcare ^*^ costs were rising more rapidly during the past decennia than gross domestic products, and this generally is considered not to be sustainable ^1^. One important explanation for these observations is Baumol’s conclusion that growth of wages in excess of productivity growth drives growth of health care expenditure ^2^. However, there is a poor correlation between health care system structures and spending with patient health outcomes ( Table 1).

**Table 1.** Comparing high income country health system resourcing and achievements (Data Source: OECD - Health at a Glance 2017 ^14^).

	OECD	US	UK	Switzerland	Australia	World
**Health Care Resources**						
Distribution of healthcare spending 2014 Public/Private	-	48%/52%	81%/17%	66%/34%	67%/33%	60%/40%
Per capita spending 2014	$ 4,003	$ 9,892	$ 4,192	$ 7,919	$ 4,708	$ 1,061
Healthcare spending as % of GDP 2016	9.0%	17.2%	9.7%	12.4%	9.6%	
Annual per capita healthcare spending increase 2003-09	3.6%	2.5%	3.9%	1.4%	2.7%	
Annual per capita healthcare spending increase 2009-16	1.4%	2.1%	0.9%	2.8%	2.7%	
Doctors/1,000 population	3.4	2.6	2.8	4.2	3.5	
Nurse/1,000 population	9.0	11.3	7.9	18.0	11.5	
Beds/1,000 population	4.7	2.8	2.6	4.6	3.8	
**Outcome of Care**						
Life Expectancy M/F	77.9/83.1	76.3/81.2	79.2/82.8	80.8/85.1	80.4/84.5	
Life Expectancy at age 65	19.5	19.3	19.7	20.9	20.9	
Ischaemic Mortality, age-standardised rate/100,000	112	113	98	78	85	
Dementia Prevalence per 1,000	14.8	11.6	17.1	17.2	14.2	
**Access to Care**						
Population covered by insurance	97.9%	90.9%	100.0%	100.0%	100.0%	
Final household consumption to cover out of pocket expenses	3.0%	2.5%	1.5%	5.3%	3.1%	
Consultations skipped due to cost - age-sex standardised rate per 100 population	10.5%	22.3%	4.2%	20.9%	16.2%	
**Outcomes of Care**						
Asthma and COPD hospital admission - Age-sex standardised rate per 100,000 population	236	262	303	138	371	
Antibiotics prescribed - defined daily dose per 1,000 population	20.6	-	20.1	-	23.4	
Acute Myocardial Infarction mortality - Age-sex standardised rate per 100,000 population	7.5	6.5	7.1	5.1	4.0	
Obstetric trauma (instrument) - Crude rate per 100 vaginal deliveries	5.7	9.6	6.8	7.4	7.2	
Foreign body left in during procedures/100,000 discharges (surgical admission method)	5.4	7.5	7.2	12.3	8.8	
Post-operative DVT or PE following hip and knee surgery/100,000 hip and knee discharges (surgical admission method)	357/301	209/294	202/316	237/339	1,113/549	
**Population Health**						
Diabetes	7.0%	10.8%	4.7%	6.1%	5.1%	
Obesity	19.4%	35.2%	26.9%	10.3%	27.9%	
Smokers, age >15	18.4%	11.4%	16.1%	20.4%	12.4%	
Alcohol consumption, age >15 in litres	9.0	8.8	9.5	9.5	9.7	
Population eating fruit daily, age >15	56.6%	57.9%	62.6%	61.5%	95.0%	
Population eating vegetables daily, age >15	59.8%	92.4%	65.5%	68.5%	99.0%	

The table highlights the differences in health system performance amongst 4 selected OECD-countries with distinctively different health system structures. Performance outcomes arises from the unique dynamic behaviors of the system, i.e. outcomes cannot be attributed to one or two specific features of the system. It also means that direct comparison of outcomes between different systems is difficult as they depend on each system’s unique characteristics and dynamics.

The relative contributions of commonly intimated factors such as scientific and technological progress in medicine and changing age demographics on healthcare expenditure and/or health system performance remain uncertain.

Major efforts to lower healthcare expenditure by applying economic principles like fundholding, limiting services, capping or bundling payments, lean management, guidelines or pay-for-performance incentives have been tried in various jurisdictions; evaluations of these interventions on overall financial burden on society and/or patient/population health outcomes remain limited and unconvincing ^1^. Economically driven initiatives demonstrably increased the administrative load of health care professionals as it has detracted them from their primary task – to expertly and professionally attend to patients care needs ^2, 3^. Studies have identified that newer technical equipment and newer drugs are two factors that unequivocally make health care more expensive ^4^. In addition many physician activities and procedures are not truly purposeful for the achievement of better health, an observation that has led to the “Choosing Wisely”movement ^5^. The relative contribution of this policy on healthcare costs and outcomes is outstanding.

**Figure 1.**
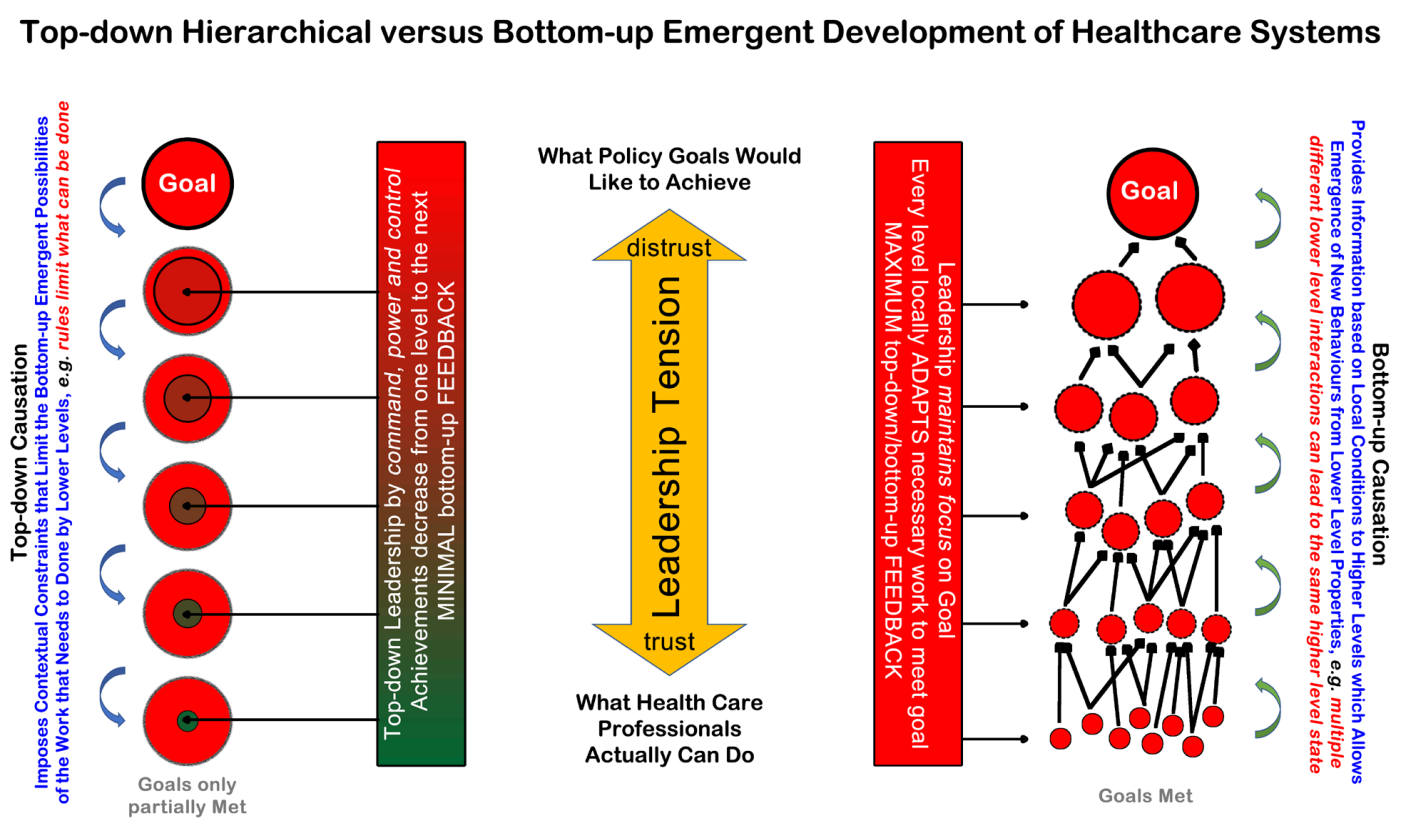
A conceptual model of the implications of top-down versus bottom-up leadership on the function of health systems. The effects of the top-down policy-driven approach on health care delivery are illustrated by the ever-decreasing size of the inner circles from one organizational level to the next where each level further constrains what the next lower level can achieve – the top-down leadership’s constraints minimize bottom-up feedback (left). The bottom-up approach is illustrated by dotted circles – to emphasize the open and adaptive nature of entities at each level- all focused on the system’s overall goal. Every higher-level circle emerges as a result of various interactions (arrows) at a lower level, resulting in the variance of characteristics and behaviors that depend on unique local circumstances. While each level shows variability in its components, each level component is the best adapted version of this level in its unique environment, and each does uniquely contribute to the achievement of the overall policy goals & settings – leadership minimizes constraints and encourages constant feedback across all levels of the system (right). Note that the complexity of a system arises from the feedback loops between top-down and bottom-up interactions across all the layers of the system. These two approaches are not mutually exclusive, rather – as the figure highlights – reflect the tension in leadership between trust (minimize constraints, maximize contextual adaptation) and distrust (maximize constraints, minimize variability). For a detailed discussion on causation in complex adaptive systems see Ellis ^8^; for a discussion on complex adaptive organizations see Laloux ^7^.

In high income countries healthcare systems generally are organized top-down. This hierarchical structure goes from the health ministry all the way down to the youngest physicians, nurses and orderlies in hospitals or physician practices ^6^. Since all coworkers must contribute according to rules from above, it is assumed that such systems lose an important part of their intrinsic motivation and productivity. Another method to organize health care would be bottom-up ^7, 8^. Such system organization implies that for each specific condition physicians and nurses who work with the patients know best how to optimally perform their work. Therefore, they are invited to first, continuously contribute to the system’s overall development and second, to adopt their own working rules, a feature that is applied to all scales of the organization ( Figure 1). It has been hypothesized that bottom-up organizations create best adapted solutions to changing problems and needs, a hypothesis supported by the experiences of the NUKA health system in Alaska ^9, 10^ and the EDARP health system in Kenya ^11, 12^ and are detailed below.

This paper sets out to explore complex adaptive system (CAS) thinking to the organization and function of healthcare systems. Complex adaptive dynamics provide the theoretical basis to the structure and function of bottom-up organizations ^13^. Initially we present the nature of a CAS as applied to healthcare including some possible perspectives for the improvement of healthcare systems in general. From this we will consider how CAS understandings may change healthcare systems and thereby benefit patients and healthcare personnel while simultaneously reducing costs.

## What is a CAS?

**Figure 2.**
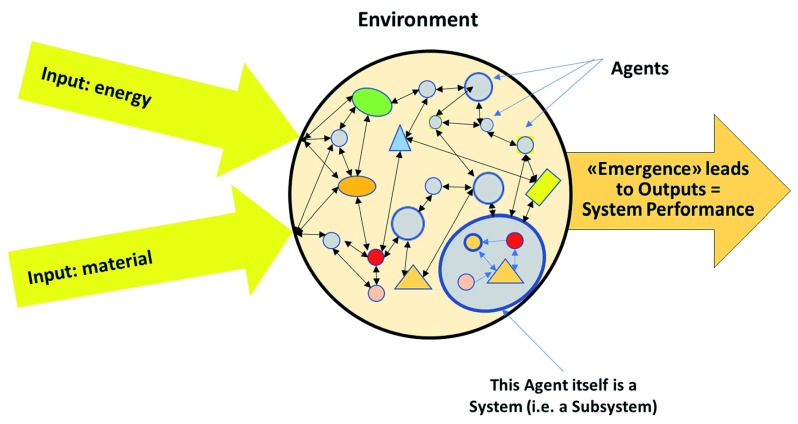
General structure of a CAS. Complex adaptive systems are open, i.e. they receive inputs from their external environment, and the interactions – especially feedback loop interactions – between its agents result in emergent outcomes that can be shared with external agents or other systems.

In general terms a CAS is an autonomously functioning open system separated from its surroundings by a fuzzy boundary, i.e. it can receive inputs from and provide outputs to its environment ( Figure 2). Its inside is composed of active parts, called agents, that continuously and spontaneously interact with each other without external control. These interactions may be simple (i.e. linear and predictable) where cause and effect are fixed, complicated (still linear and predictable) where a particular cause results in a particular outcome (often with a delay in time or place), complex (i.e. nonlinear) where cause and effect are perceivable but not precisely predictable, or chaotic (i.e. unrelated) where no cause and effect relationship is evident. Interactions among the agents of a CAS result in feedback, and feedback drives the emergent behavior of the system as a whole ^13^.

Two additional features contribute to the complex adaptive dynamics of a CAS. Firstly, many agents are CAS in their own right, i.e. they constitute subsystems, and vice versa, each CAS itself is part of a larger supra-system. This nested nature of CAS results in a hierarchical layering where higher layer supra-systems “constrain” the potential “bottom-up” emergent behavior of lower layer subsystems ^8^. Secondly, the interdependencies between the nested hierarchical structure and the dynamics resulting from the opposing forces of “top-down” constraints and “bottom-up” emergent potentials makes CAS “stable and resilient” in constantly fluctuating environments (i.e. CAS are in a non-equilibrium state). Internal and external perturbations into a non-equilibrium system contribute to its emergence over time, and this may have no influence or enhance or diminish the system’s overall performance and stability. This means that a CAS can evolve in response to needs of its surroundings. Rarely is the input into a system large enough to cause a complete and/or abrupt system change.

## The healthcare system is a “socially constructed” CAS

**Figure 3.**
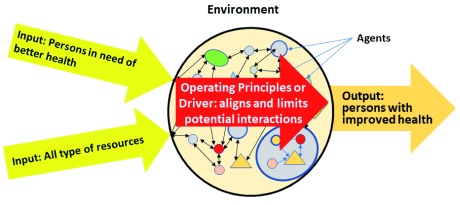
General structure of the healthcare system. The driver of the health system – resulting from its agreed purpose, goals and values – align and limit the potential interactions of its agents in response to diverse inputs. These constraints “determine” the potential outcomes the health system can deliver, both in terms of health outcomes for the patients treated and the economic and resource costs associated with the service delivery.

Healthcare systems are “organizational systems”, thus they are socially constructed. An organizational CAS emerges based on purpose, goal and value propositions that give rise to its operating principles or driver. Combined they provide the “top-down” constraints that limit the “bottom-up” emergent possibilities of its agents at the various levels within the healthcare system ( Figure 3).

Besides of health professionals and support workers a health system’s agents also include - amongst others - politicians, administrators, pharmaceutical organizations, devise makers and insurance companies. System “inputs” in the first instance consist of persons in need of better health. Other important inputs are resources like new knowledge, technologies, finances, drugs and technical equipment, etc. The overall performance of the CAS results in emergent “outputs”, i.e. “persons with improved health”.

A healthcare system’s driver “focuses or directs” the activities of its agents. It tends to support influences that are consistent with its established purpose, goals and values. It thereby allows the emergence of appropriate structures and functions necessary for its overall performance. Thus, a health system’s driver may allow changes to the structure, e.g. the addition of a new health service division (structural change) or the implementation of a new service delivery approach (functional change). Success requires bottom-up adoption as the “current successful drivers” of a CAS tend to strongly resist top-down “instructions” that contradict, restrain or impede the status quo.

## The role of governance - Top-down versus bottom-up

A socially constructed CAS functions based on its socially constructed driver arising from the system’s definition of its purpose, goals and values statement. The driver ultimately can be “controlled” – in a governance sense – top-down “bureaucratic”, or bottom-up “grass-roots”.

The schematic comparison depicted in Table 2 reveals fundamental differences between these two types of governance. A traditional organizational system uses hierarchy and manages the organizations top-down. Motivation of coworkers is extrinsic, induced by command and control and human relationships are contractual. Superiors focus on the efficiency of the system and evaluate whether or not the activities are appropriate (process oriented). In contrast, governance in a complex adaptive organizational system is based on heterarchy and personal leaderships. The structure is bottom-up self-organizational. Motivation is intrinsic by identification with the purpose, goals and values of the organization. Human relationships are based on personal commitment and the focus of employees is problem-oriented. To supervise the organization the leadership assesses the outcome (outcomes oriented).

**Table 2.** Comparison of governance in traditional and complex organizations (adapted from Rouse ^16^)

	Traditional organizational system	Complex adaptive organizational system
***Organization***	Hierarchy	Heterarchy
***Roles***	Management	Leadership
***Design***	Top-down organization	Bottom-up self-organization
***Motivation***	Command and control	Sense, purpose and norms
***Relationships***	Contractual	Personal commitment
***Focus***	Efficiency	Problem-orientation
***Measurement***	Activities	Outcomes

The principles of leadership between the two types of governance are fundamentally different. Leadership in hierarchical systems relies on power, command and control, whereas leadership in heterarchical system is based on collaboration, respect, learning from each other and measuring of outcomes ^7^.

## From theory to first-hand experience

Observation would suggest that it is always “easier” to live with the imperfection of the status quo and to fiddle with its imperfection at the margins – despite all the talk about the failing health systems around the world. The top-down improvement efforts of the past 30+ years have little to show for. However, there are some notable examples that support the hypothesis that bottom-up approaches create organizations that deliver highly adapted solutions to the changing problems and needs of their patients/communities in a more efficient and cost-effective way.

### The needs of the patient come first – the 100-year-old driver of the Mayo Clinic

The Mayo brothers have been the first to organize their hospital-based health care around a system driver, codified in the motto ^Ɨ^ of “ *The needs of the patient come first*.” ^15^.

To prevent monetary inducements influencing clinical decision-making the Mayo founders took the “radical step” to employ all physicians (and all other staff) on specialty adjusted fixed salaries. The hierarchy among physicians is flat, and accepted patients (based on a “medical needs assessment”) are treated irrespective of their capacity to pay. Importantly the clinic’s medical ethics are cultivated continuously and are self-reinforcing.

For many years the Mayo Clinic has now remained the number one healthcare organization for patient care in the United States. Careful consideration by its leadership of the sense or purpose of healthcare, its financing and physician ethics, i.e. the “driver” of the Mayo Clinic, have maintained its longstanding success in a constantly changing health care envirionment ^15^. Today the Mayo Clinic is regarded as the best practice model of health service delivery in the US in a primarily tertiary oriented healthcare organization – achieving great health outcomes in a most cost-effective and efficient way.

### Re-defining the driver of a healthcare system – the NUKA health system

An inadequate centrally controlled *American Indian Health Service* morphed into the highly functioning NUKA health system as a result of a bottom-up change to the system’s drivers. Alaskan native people realized a bottom-up customer owned system oriented toward physical, mental, emotional and spiritual wellness through community and interprofessional cooperation. The change of their health system’s driver to embrace “shared responsibility, commitment to quality and family wellness” achieved a healthcare service that “finally” meets its patients’ and community’s needs and aspirations. Ongoing collaboration ensures that the system remains responsive to the community’s evolving requirements as well as medical progress, something that the previous top-down organization by a Washington-based government bureaucracy could not achieve ^9, 10^.

As health systems are constructed socially “finding the right driver” – as illustrated by the Alaskan native people’s approach – can lead to the emergence of a health system that appropriately meets its users’ needs. When bottom-up “improvement of performance” is allowed to drive a healthcare system it can evolve to meet the system’s overarching goals and purposes while locally delivering amongst others patient centered care based on scientific progress and technological advances.

Besides of being aligned with their patients’ needs and having achieved better health outcomes, the NUKA health system approach has also demonstrated that it has achieved these outcomes at lower costs ^10^.

### The Emergence of a health service driver – EDARP-Kenya, Buurtzorg-The Netherlands and the village health service in Odisha-India

***Eastern Deanery AIDS Relief Program ( EDARP).*** The Eastern Deanery AIDS Relief Program (EDARP) is an example that demonstrates how a “clearly defined” driver can create a community-based health service “de-novo”. Initially the program solely aimed to relieve the suffering of dying AIDS patients. However, the community health workers involved in the care of these patients identified many additional interconnected needs – at the personal, social and community levels – that resulted in the emergence of a community led, community delivered health and social service network for a Nairobi slum district that has dramatically improved health outcomes at the personal and community levels for this disadvantaged population ^11, 12^.

***Buurtzorg – Dutch for “neighborhood care”.*** A fascinating example of bottom up governance has been realized in the Netherlands by the project “Buurtzorg ^‡^” ^17^. This is a pioneering organization established in 2006: A nurse-led bottom-up model of holistic care assumes responsibility for ambulatory nursing in the communities. It not only revolutionized community care, but client satisfaction rates are the highest of any health care organization; staff commitment and contentedness is superior. Ernst & Young documented savings to the Dutch health care system of around 40%, if all care would be provided this way ^18^.

***A care system for Indigenous people in Odisha-India.*** Another example showing a bottom-up success concerns health services in villages of recently settled indigenous people in Odisha, India. These villages received top down basic health care by the Indian government. Yet, many villagers refused vaccinations of their children and used supplied mosquito nets just for fishing. When members of an NGO that had cared for the development of these villages explained the Meikirch model of health to the inhabitants their health-related behavior improved markedly. Ninety percent of informed villagers washed their hands before meals, while in control villages without teaching only 41% did it. Eighty percent of households had latrines in comparison to 42% in control villages, and 98% of children were vaccinated compared to 58% in control villages. These results confirm that indigenous villagers do not respond satisfactorily to “gifts” from the government, but they can understand teaching about health and correspondingly change their behavior ^19^.

## What has been learnt

A complex adaptive organization is in constant flux responding to diverse external inputs that challenge its internal structures and dynamics. Lived purpose, goals and values statements are the basis for a system’s driver that ultimately governs the behavior of complex adaptive organizations and ensures a level of dynamic stability. Prevailing top-down organizational leadership, based on command, power and control, invariably results in limited emergent staff engagement stifling staff morale, and in turn diminishes their creativity and productivity. Alternatively, organizations can adopt a bottom-up management approach fostering collaboration, respect and learning, making the organization more resilient.

Bottom-up minded complex adaptive organizations usually have a well-defined purpose, clearly discernable goals and transparent values that together give rise to the system’s driver. Three features underpin an effective driver of a bottom-up health system or service: a focus on health, minimizing financial collusion, and adhering to Hippocratic norms of the medical professions ^13^.

The difference between these two leadership mindsets is the nature of the constraints created – the more restrictive they are the more they limited what staff at each organizational level can do. Neither leadership style changes the fact that leaders are ultimately responsible for their organization’s performance and achievements. 

### Knowing the purpose is all important

Restoring or improving a person’s health is the core purpose of health care delivery. Hence, “improvement of health” must be part of the driver for health care, although “improved health” until now could only be understood intuitively and has only tacitly shaped patient/physician interactions.

The lack of a precise definition of health, and as a corollary disease, has remained an important defect. Without a clear understanding of the meaning of health healthcare systems cannot offer an enabling vision to its staff and their patients, and unsurprisingly allowed arbitrariness in decision-making and management based on economic or personal interests. Over the last 5 years, science based models that define health and disease have emerged – the first being the Meikirch-model ^20, 21^ whose fundamental tenet has recently been corroborated by a multi-disciplinary group collaboration demonstrating the interconnectedness and interdependence of external and internal variables on the dynamic state of health ^22^. The purpose of healthcare therefore no longer remains intuitive and difficult to communicate. It can now be analyzed and expressed explicitly. As a result, it is possible to devote the attention of physicians, nurses and other health care workers to each individual patient’s existential health needs. Today we are able to “scientifically” reconnect with our predecessors who devoted their lives as nurses or physicians to this fulfilling task with financial modesty and much personal satisfaction.

### Financial priorities have reframed the focus of health systems

In recent decades health professionals’ attitudes and approaches to health care delivery have become compromised by focusing on profit maximization. As a result, financial interests have distorted health care away from its central mission: Patients are no longer certain that they are advised only according to their personal health needs rather than being seen as the means to achieve the financial interests of institutions or some of their health professionals.

Today health professionals struggle with the tensions arising from their core duty of meeting the health needs of their patients and the pressures exerted on them to practice within the limited financial resources provided to them. While there clearly is a limit to resourcing health care systems one nevertheless has to acknowledge that almost all medical decision-making has a wide margin of discretion. Organizational leaders easily (and frequently) modified discretional decision-making applying external forces, like financial incentives, nudging and competition ^23, 24^.

Unquestionably though, *financing of a complex adaptive healthcare organization* should have no other purpose than to provide adequate resources to deliver needed health care services to its patients/communities. Unfortunately, in the past decades financial pressures have been applied increasingly and widely. They have been used to increase physicians’ “productivity”, to modify their behaviors in relation to diagnostic and therapeutic approaches as well as for the specific purpose to reduce overall healthcare costs.

Yet, financially driven interventions tend to disregard the best interest of patients, and have failed to diminish costs, but – as an unintended consequence – have resulted in delayed access to healthcare and increased costs ^25^. In most cases financial incentives only temporarily changed incentivized clinician behaviors but more importantly they damaged health system design and/or health outcomes ^26, 27^.

In addition, excessive advertising of technologies to attract patients to specific institutions also have influenced and deteriorated patient care and increased waste ^28, 29^. Simultaneously excessive administration and prolonged working hours proved harmful for health service personnel ^29, 30^. Although originally intended to reduce healthcare costs, top-down politically or economically motivated measures have augmented administrative workloads and resulted in increasing frustration followed by an exodus of physicians and nurses ^29^.

The sum of these observations confirms that first priority cost-containment as a driver of a healthcare system by necessity leads to failure. Health system financing is important, but it must serve medical care delivery that improves patients’ health. It must not direct it. That said, there equally is no place for waste in health care – whilst highest quality at the lowest possible cost remains a priority, improvement of a patient’s health must be respected as the first priority.

### Hippocratic norms remain central

Since antiquity *ethical norms* have played important roles in all spheres of life. Confucius’ golden rule is a most famous example: “ *Never impose on others what you would not choose for yourself.*” He lived in China from 551 to 479 AC. Around the same time, at the other side of the world, the Hippocratic oath emerged as the guiding frame for the conduct of physicians. Since 1948 it has been periodically updated by the World Medical Association and renamed “Declaration of Geneva”. The most recent revision in October 2017 ^31^ begins with this affirmation: “ *As a member of the medical profession I solemnly pledge to dedicate my life to the service of humanity. The health and wellbeing of my patient will be my first consideration.*” Importantly, the declaration does not concern itself with the income of physicians, however, by implication may allude to a physician’s financial responsibilities in a later statement: *“I will practice my profession with conscience and dignity and in accordance with good medical practice.”* Of note, the Geneva Declaration expresses fidelity toward patients and the physician’s personal integrity while tacitly acknowledging economic and financial concerns.

## Conclusions: Respecting the nature of complex adaptive health systems achieves better health outcomes at lower cost

Healthcare systems are socially constructed complex adaptive organizations. As other complex adaptive systems they are driven by three components, their explicitly expressed purpose, their goals and their values. First examples of CAS are the Buurtzorg ambulatory nursing, the NUKA or the EDARP health systems. These organizations fulfill their purpose by having created “loose enough constraints” that foster bottom-up emergent behaviors enabling their staff to adaptively respond to changing patient needs and economic constraints. 

These deliberations and examples demonstrate that it is indeed possible to develop and adjust the driver of a CAS in the combined best interest of patients and society. Successful complex adaptive organizations have distributed leadership that fosters collaborative learning to adapt to the changing needs of its patients. For the society they promise to be more effective and more efficient at reduced costs. 

## Data availability

No data is associated with this article.

## Footnotes

*Healthcare refers to the institution, health care to the deliverables of its agents

ƗAn interesting example of attention to the driver of healthcare is given by the Mayo Clinic in Rochester Minnesota, USA. In 1910, William James Mayo, M.D., delivered the commencement address at Rush Medical College in Chicago declaring that: "The best interest of the patient is the only interest to be considered, and in order that the sick may have the benefit of advancing knowledge, a union of forces is necessary." This ethos ever since has driven the Clinic’s approach to patient care.

‡translates as “neighborhood care”

## References

[1] WHO: WHO New Perspectives on Global Health Spending for Universal Health Coverage European Region Health Expenditure Dashboard, 2000–2015. In: Global health spedning. Reference Source

[2] Hartwig J Sturm JE: Robust determinants of health care expenditure growth. *Appl Econ.* 2014;46(36):4455–74. 10.1080/00036846.2014.964829

[3] Faller MS Gates MG Georges JM: Work-related burnout, job satisfaction, intent to leave, and nurse-assessed quality of care among travel nurses. *J Nurs Adm.* 2011;41(2):71–7. 10.1097/NNA.0b013e3182059492 21266885

[4] Bodenheimer T: High and rising health care costs. Part 2: technologic innovation. *Ann Intern Med.* 2005;142(11):932–7. 10.7326/0003-4819-142-11-200506070-00012 15941701

[5] Lehmann C Berner R Bogner JR: The "Choosing Wisely" initiative in infectious diseases. *Infection.* 2017;45(3):263–8. 10.1007/s15010-017-0997-0 28290130

[6] Uvhagen H Hasson H Hansson J: Leading top-down implementation processes: a qualitative study on the role of managers. *BMC Health Serv Res.* 2018;18(1):562. 10.1186/s12913-018-3360-y 30021569 PMC6052667

[7] Laloux F: Reinventing Organizations, A Guide to Creating Organizations Inspired by the Next Stage of Human Consciousness. Knowledge Partners, Maharashtra India,2018.

[8] Ellis GF: Top-down causation and emergence: some comments on mechanisms. *Interface Focus.* 2012;2(1):126–40. 10.1098/rsfs.2011.0062 23386967 PMC3262299

[9] Gottlieb K: The Nuka System of Care: improving health through ownership and relationships. *Int J Circumpolar Heal.* 2013;72(1):21118. 10.3402/ijch.v72i0.21118 23984269 PMC3752290

[10] Collinss B: Intentional whole health system redesign: Southcentral Foundation’s ‘Nuka’ system of care. *Kings Fund.* 2015 Reference Source

[11] Njoroge A Cassidy S Williams V: Making patient-centred care a reality in the slums of eastern Nairobi. *Int J Tuberc Lung Dis.* 2013;17(10 Suppl 1):5–8. 10.5588/ijtld.13.0186 24020594

[12] Sturmberg JP Njoroge A: People-centred health systems, a bottom-up approach: where theory meets empery. *J Eval Clin Pract.* 2017;23(2):467–73. 10.1111/jep.12540 27062608

[13] Sturmberg JP: Health System Redesign.2018 10.1007/978-3-319-64605-3

[14] OECD: Health at a Glance 2017: OECD Indicators. OECD Publishing, Paris,2017 10.1787/health_glance-2017-en

[15] Berry L Seltman K: Management Lessons from Mayo Clinic: Inside One of the World’s Most Admired Service Organizations. McGraw-Hill, New York,2008 Reference Source

[16] Rouse WB: Health Care as a Complex Adaptive System: Implications for Design and Management. *Bridg.* 2008;38:17–25. Reference Source

[17] Monsen KA de Blok J: Buurtzorg: Nurse-Led Community Care. *Creat Nurs.* 2018;24(1):112–7. 10.1891/1078-4535.19.3.122 29669644

[18] Buurtzorg. Reference Source

[19] Samal S Mohanti D Born E: Teaching of health with the Meikirch model to indigenous people improves their health-supporting behavior: A pilot study. *Med J DY Patil Univ.* 2017;10(1):17–20. 10.4103/0975-2870.197902

[20] Bircher J Kuruvilla S: Defining health by addressing individual, social, and environmental determinants: new opportunities for health care and public health. *J Public Health Policy.* 2014;35(3):363–86. 10.1057/jphp.2014.19 24943659 PMC4119253

[21] Bircher J Hahn EG: Understanding the nature of health: New perspectives for medicine and public health. Improved wellbeing at lower costs [version 1; peer review: 2 approved]. *F1000Res.* 2016;5:167. 10.12688/f1000research.7849.1 27134730 PMC4837984

[22] Sturmberg JP Picard M Aron DC: Health and Disease-Emergent States Resulting From Adaptive Social and Biological Network Interactions. *Front Med (Lausanne).* 2019;6:59. 10.3389/fmed.2019.00059 30984762 PMC6447670

[23] Lagarde M Blaauw D: Physicians' responses to financial and social incentives: A medically framed real effort experiment. *Soc Sci Med.* 2017;179:147–59. 10.1016/j.socscimed.2017.03.002 28279924

[24] Doran T Maurer KA Ryan AM: Impact of Provider Incentives on Quality and Value of Health Care. *Annu Rev Public Health.* 2017;38:449–65. 10.1146/annurev-publhealth-032315-021457 27992731

[25] Haren MC McConnell K Shinn AF: Increased Patient Cost-Sharing, Weak US Economy, and Poor Health Habits: Implications for Employers and Insurers. *Am Heal Drug Benefits.* 2009;2(3):134–41. 25126283 PMC4106557

[26] Alshamsan R Majeed A Ashworth M: Impact of pay for performance on inequalities in health care: systematic review. *J Heal Serv Res Policy.* 2010;15(3):178–184. 10.1258/jhsrp.2010.009113 20555042

[27] Ryan AM Krinsky S Kontopantelis E: Long-term evidence for the effect of pay-for-performance in primary care on mortality in the UK: a population study. *Lancet.* 2016;388(10041):268–74. 10.1016/S0140-6736(16)00276-2 27207746

[28] Berwick DM Hackbarth AD: Eliminating waste in US health care. *JAMA.* 2012;307(14):1513–6. 10.1001/jama.2012.362 22419800

[29] Alexander D: How Do Doctors Respond to Incentives? Unintended Consequences of Paying Doctors to Reduce Costs. *Work Pap Ser WP-2017–9, Fed Reserv Bank Chicago.* 2017 Reference Source

[30] Wehkamp KH Heinz N: Ökonomisierung patientenbezogener Entscheidungen im Krankenhaus. *Dtsch Arztebl.* 2017;114(47):797–804. 10.3238/arztebl.2017.0797

[31] World Medical Association. Declaration of Geneva.2017 Reference Source 10.4314/ahs.v17i4.30 PMC5870282 29937893

