# Supplementary material for: Better and fulfilling healthcare at lower costs: The need to manage health systems as complex adaptive systems
Source: F1000Res. 2019 Jun 5;8:789. [Version 1] doi: 10.12688/f1000research.19414.1 (PMC6900806; doi:10.12688/f1000research.19414.1)
Supplement: Peer review history

# Peer review: Reviewer response for version 1

**DOI:** 10.5256/f1000research.21281.r57136

**Published:** 2019-12-06

**Authors:** Wikum Jayatunga

**Affiliations:** 1 Institute of Health Informatics, University College London, London, UK

This is a well-written opinion article of a topic of significant topical relevance as many developed health systems grapple with the challenges of growing healthcare needs with finite resources. Increasingly complexity science is being used to understand the behaviour and performance of healthcare systems and their agents, and this paper makes a convincing case for the importance of values/purpose/goals (which are sometimes neglected over financial/economic concerns).

 The logic and flow of the argument is sound and well-supported by the cited literature. While the benefits of bottom-up approaches to leadership are the main thrust of the paper, with interesting case studies used as examples, the paper is measured in its conclusions: recognising that the top-down and bottom-up approaches are not mutually exclusive.

 I suggest revision of the following sentence in the section 'From theory to first-hand experience'. "Observation would suggest that it is always “easier” to live with the imperfection of the status quo and to fiddle with its imperfection at the margins." As the writing style feels out of place, although this is only a minor point.

I confirm that I have read this submission and believe that I have an appropriate level of expertise to confirm that it is of an acceptable scientific standard.

# Peer review: Reviewer response for version 1

**DOI:** 10.5256/f1000research.21281.r52863

**Published:** 2019-08-27

**Authors:** Jochen Hartwig

**Affiliations:** 1 KOF Swiss Economic Institute, ETH Zurich, Zurich, Switzerland; 2 Department of Economics and Business Administration, Chemnitz University of Technology, Chemnitz, Germany

The authors argue in favor of organizing healthcare systems bottom-up instead of top-down and discuss several examples of successful implementation of that principle around the world.

 The contribution is an ‘opinion article’. Whether or not I agree with the authors´ opinion is not relevant. In my view, the article is a stimulating contribution to the debate on healthcare systems reform and should be indexed as such.

 For a final version of the paper I suggest explaining "the Meikirch model" on p. 7 where it is first mentioned. In the fourth paragraph on the left hand side of p. 8 I think it should read 'limit' instead of 'limited'. Finally, I recommend dropping the first sentence on the right hand side of p. 8 because, in my view, it sounds a bit too lofty for a scholarly article.

I confirm that I have read this submission and believe that I have an appropriate level of expertise to confirm that it is of an acceptable scientific standard.
